# Study on the Tribological Properties of DIN 16MnCr5 Steel after Duplex Gas-Nitriding and Pack Boriding

**DOI:** 10.3390/ma17133057

**Published:** 2024-06-21

**Authors:** Rafael Carrera Espinoza, Melvyn Alvarez Vera, Marc Wettlaufer, Manuel Kerl, Stefan Barth, Pablo Moreno Garibaldi, Juan Carlos Díaz Guillen, Héctor Manuel Hernández García, Rita Muñoz Arroyo, Javier A. Ortega

**Affiliations:** 1 Departamento de Ingeniería Industrial y Mecánica, Escuela de Ingeniería, Universidad de las Américas Puebla, Ex-Hacienda Santa Catarina Mártir S/N, Puebla 72810, Mexico; rafael.carrera@udlap.mx (R.C.E.); pablo.moreno@udlap.mx (P.M.G.); 2 Centre of Materials Engineering, Heilbronn University, Max-Planck-Straße 39, 74081 Heilbronn, Germany; marc.wettlaufer@hs-heilbronn.de (M.W.); makerl@stud.hs-heilbronn.de (M.K.); stefan.barth@hs-heilbronn.de (S.B.); 3 Innovabienestar de México, SAPI de CV, Ciencia y Tecnología No. 790, Col. Saltillo 400, Saltillo 25290, Mexico; jcarlos@comimsa.com (J.C.D.G.); hmanuelhdz@comimsa.com (H.M.H.G.); 4 Facultad de Sistemas, Universidad Autónoma de Coahuila, Carr. A México km. 13, Saltillo 25280, Mexico; munoz.r@uadec.edu.mx; 5 Department of Mechanical Engineering, The University of Texas Rio Grande Valley, 1201 West University Drive, Edinburg, TX 78539, USA; javier.ortega@utrgv.edu

**Keywords:** gas-nitriding, pack boriding, DIN 16MnCr5 steel, tribology, microstructure, mechanical properties

## Abstract

DIN 16MnCr5 is commonly used in mechanical engineering contact applications such as gears, joint parts, shafts, gear wheels, camshafts, bolts, pins, and cardan joints, among others. This study examined the microstructural and mechanical properties and tribological behavior of different surface treatments applied to DIN 16MnCr5 steel. The samples were hardened at 870 °C for 15 min and then quenched in water. The surface conditions evaluated were as follows: quenched and tempered DIN 16MnCr5 steel samples without surface treatments (control group), quenched and tempered DIN 16MnCr5 steel samples with gas-nitriding at 560 °C for 6 h, quenched and tempered DIN 16MnCr5 steel samples with pack boriding at 950 °C for 4 h, and quenched and tempered DIN 16MnCr5 steel samples with duplex gas-nitriding and pack boriding. Microstructure characterization was carried out using metallographic techniques, optical microscopy, scanning electron microscopy with energy-dispersive spectroscopy, and X-ray diffraction. The mechanical properties were assessed through microhardness and elastic modulus tests using nanoindentation. The tribological behavior was evaluated using pin-on-disc tests following the ASTM G99-17 standard procedure under dry sliding conditions. The results indicated that the surface treated with duplex gas-nitriding and pack boriding exhibited the highest wear resistance and a reduced coefficient of friction due to improved mechanical properties, leading to increased hardness and elastic modulus.

## 1. Introduction

DIN 16MnCr5 is widely used in industry for a variety of contact applications, such as gears [1,2], gear wheels [3,4], shafts [5,6], camshafts [7,8], bolts [9,10], and pins [11,12]. One of the significant challenges in today’s industry is modifying and enhancing metallic surfaces for contact applications used in critical machine parts. Surface engineering aims to enhance material performance by improving mechanical properties such as increased surface hardness, toughness, and tribological behavior. This improvement helps reduce wear and friction and enhances the components’ performance when interacting with other contact surfaces.

Modifying the steel surface using different surface hardening techniques such as nitriding and boriding can enhance the mechanical and tribological properties. Nitriding, in particular, is widely utilized as a diffusion hardening method for structural steels [13]. In this sense, several studies have focused on enhancing the mechanical properties of DIN 16MnCr5 steel surface materials by using different nitriding processes such as plasma, salt bath, and gaseous nitriding. Cieslik et al. [14] conducted a study on the plasma nitriding treatment process for DIN 16MnCr5 steel. They investigated the impact of an additional diffusion stage, using different gas compositions of nitrogen (N_2_) and hydrogen (H_2_), on the compound layer thickness and the hardness profile of the steel. Additionally, Esfahani et al. [15] analyzed the effect of quenched and tempered AISI 5115 (DIN 16MnCr5) steel with plasma-nitrided and nitrocarburized in N_2_ atmospheres balanced with CO_2_ and H_2_ gases to enhance the corrosive properties. In the salt bath nitriding process for DIN 16MnCr5 steel, Wong-Ángel et al. [16] used a mixture of 60–70 wt.% sodium cyanide (NaCN) and 30–40 wt.% potassium cyanide (KCN). They preheated the component to be nitrided to 420 °C and then immersed it in a molten salt bath at 580 °C for 6 h, resulting in improved tribological performance. Another study on the salt bath nitriding process for DIN 16MnCr5 steel was conducted by Arunkumar et al. [5]. They determined the appropriate heat treatment process parameters for hardening the steel, which involved heating to 860 °C to 930 °C in a powder/salt bath and subsequent cooling in oil/a hot bath at 160 °C to 250 °C. The gaseous nitriding process for DIN 16MnCr5 steel employed ammonia (NH_3_) or nitrogen (N_2_) and additional gases such as argon (Ar), hydrogen (H_2_), or air. Vivek [17] studied how carbonitriding with propane and ammonia gases affected the mechanical properties and hardness of DIN 16MnCr5. Similarly, Arumparithy et al. [18] combined carburizing and nitriding using CO_2_ and ammonia to enhance the hardness and wear resistance of DIN 16MnCr5 steel. Caliari et al. [19] investigated nitriding on DIN 16MnCr5 steel using ammonia (NH_3_) (50%), CO_2_, and N_2_ for 4 h, resulting in improved mechanical properties and increased hardness. Caliari et al. [20] also examined the N_2_ diffusion depth of DIN 16MnCr5 steel, which led to increased internal and external hardness. Additionally, Khusainov et al. [21] looked at how the hydrogen content in the working gas affected the growth kinetics of the hardened layer during ion nitriding of DIN 16MnCr5.

An alternative method of enhancing mechanical and tribological properties involves a thermochemical treatment called pack boriding. This process entails placing the sample in a sealed container filled with a boron powder mixture. The boron atoms diffuse into the surface of the workpiece, forming borides with the base metal [22]. Some research has indicated that the pack boriding process can enhance the mechanical properties of DIN 16MnCr5 steel surface materials. Calik et al. [23] conducted a study on the impact of boronizing kinetics on the structure and mechanical properties of DIN 16MnCr5 steel, leading to increased hardness, yield strength, and ultimate tensile strength. Additionally, Calik et al. [24] examined the effects of interrupting the boriding process after 5 h on the microstructure and mechanical properties of 16MnCr5 steels, resulting in increased surface hardness. Kováč et al. [25] investigated the influence of the boronizing process on the wear properties of 16MnCr5 steels, observing increases in hardness and wear resistance. Moreover, Drajewicz et al. [26] analyzed the microstructure within the diffusion pack boriding process, showing the potential to improve mechanical properties. The impact of temperature and duration of boriding on the nanohardness and modulus of elasticity of DIN 16MnCr5 steel was studied by Calik et al. [27].

Although different studies have been conducted on DIN 16MnCr5 steel to determine its mechanical and tribological properties using diffusion methods such as nitriding and boriding, the effect of duplex nitriding and boriding methods on these properties has not been found in the literature. This study aims to evaluate the microstructural and mechanical properties and tribological behavior of duplex gas-nitriding and pack boriding processes applied to DIN 16MnCr5 steel. For this study, all samples underwent hardening and quenching in water. The conditions assessed included quenched and tempered DIN 16MnCr5 steel samples without surface treatments (used as control), gas-nitriding treatment, pack boriding treatment, and combined gas-nitriding and pack boriding of quenched and tempered DIN 16MnCr5 steel. The nitriding, boriding, and combined nitriding–boriding layers formed were examined using metallographic techniques. The mechanical properties (including microhardness and elastic modulus) of the control, nitriding, boriding, and combined layers formed on the DIN 16MnCr5 steel were determined through nanoindentation tests. The tribological behavior was evaluated through pin-on-disc testing under dry sliding conditions using an alumina pin in accordance with ASTM G99-17 standard [28]. Thus, the duplex gas-nitriding and pack boriding layers formed on DIN 16MnCr5 steel could have the potential to enhance tribological behavior in mechanical engineering contact applications.

## 2. Materials and Methods

### 2.1. Gas-Nitriding and Pack Boriding Processes

**Figure 1:**
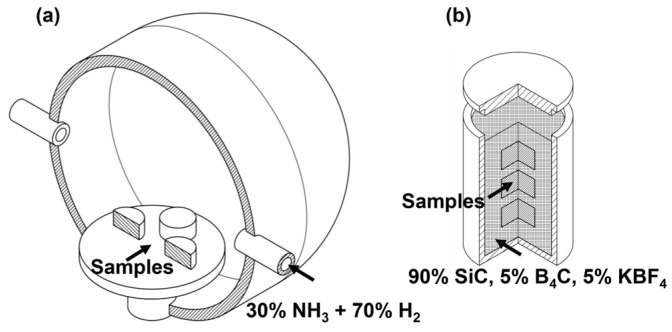
Schematic of duplex surface treatment of (**a**) gas-nitriding and (**b**) pack boriding thermochemical treatment.

A commercial rod made of AISI 5115 steel was used as the base material in this experimental procedure. It was sectioned to obtain samples with a diameter of 20 mm and a thickness of 6 mm. All samples were hardened at 870 °C for 15 min and then quenched in water. The surface conditions evaluated were as follows: quenched and tempered DIN 16MnCr5 steel samples without surface treatments (used as control), quenched and tempered DIN 16MnCr5 steel samples with gas-nitriding under 560 °C for 6 h, quenched and tempered DIN 16MnCr5 steel samples with pack boriding thermochemical treatment carried out at 950 °C for 4 h, and duplex gas-nitriding and pack boriding of quenched and tempered DIN 16MnCr5 steel, as shown in Figure 1. The parameters for the thermal, gas-nitriding, and pack boriding treatments are provided in Table 1.

The chemical composition of commercial DIN 16MnCr5 steel (ABRAMS Industries Inc., Bolingbrook, IL, USA, AISI 5115—DIN 16MnCr5 steel) used in this study is presented in Table 2.

### 2.2. Characterization

The microstructure characterization of DIN 16MnCr5 steel was performed using scanning electron microscopy (SEM), Tescan Mira 3 (TESCAN GROUP, a.s., Kohoutovice, Brno, Czech Republic); energy-dispersive X-ray spectroscopy (EDS), Bruker (Bruker, Hamburg, Germany) was performed to determine the semi-quantitative chemical composition mapping of the layered samples. The metallographic phases before and after surface treatment were determined by an X-ray diffraction analysis (XRD), Phillips X’Pert 3040, Bragg-Brentano (PANALITICAL, Great Malvern, UK) over a 2-theta range from 30 to 90 degrees by using Cu-Kα radiation at 25 kV–30 mA. Profilometer Mitutoyo SJ-410 (Mitutoyo Corporation, Kawasaki, Japan) was used to analyze the transverse wear tracks.

### 2.3. Mechanical and Tribological Properties

The microhardness HV was measured using a microdurometer micromet 6010 (BUEHLER, Shanghai, China) using a 0.1 kg load and 15 s hold time. The results were calculated by computing the average value of the microhardness after five measurements at each depth. The microhardness and elastic modulus were determined by nanoindentation testing with an RTec Instrument (RTEC-INSTRUMENTS Inc., San Jose, CA, USA) using 50 mN load and 12 s hold time.

The tribological performance of four groups with two repetitions of DIN 16MnCr5 steel was evaluated using a pin-on-disc tests tribometer TBR (Anton Paar, Ostfildern-Scharnhausen, Germany) following the procedure of ASTM standard G99-17 using 6 mm pins of hard metal (WC) under dry sliding conditions. A group of untreated DIN 16MnCr5 steel samples were tested as a control. The tribological behavior of the three groups (gas-nitriding treatment, pack boriding treatment, and duplex gas-nitriding and pack boriding of quenched and tempered DIN 16MnCr5 steel) was determined. The tribological parameters used in the test are summarized in Table 3.

## 3. Results and Discussion

### Microstructure Analyses

**Figure 2:**
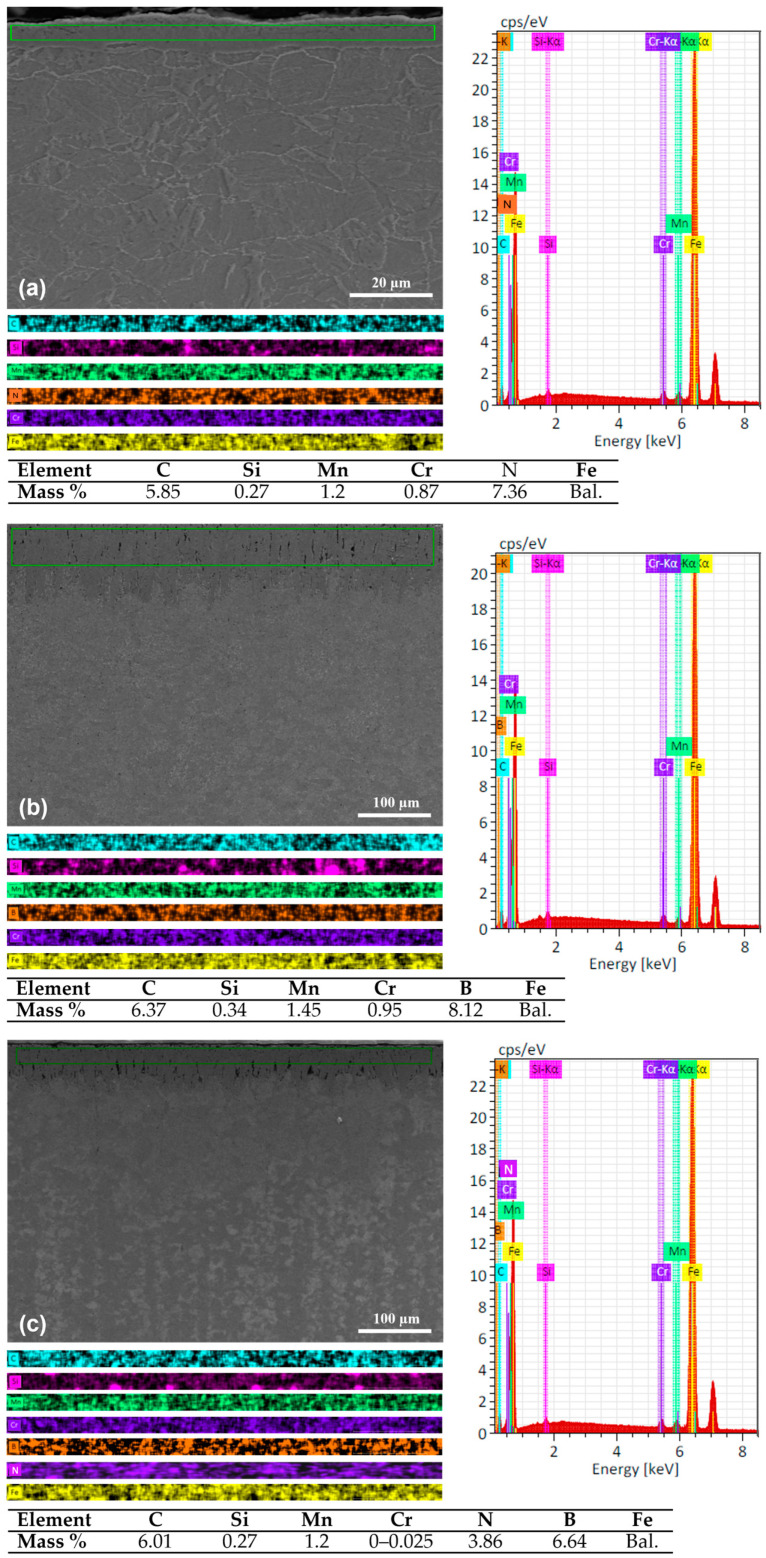
SEM micrographs with secondary electron detector and chemical composition mapping of layers for (**a**) gas-nitriding, (**b**) pack boriding, and (**c**) duplex nitriding and boriding.

The microstructure micrographs of the cross-section and formed layers—including the elemental mapping of layers formed by nitriding, boriding, and boriding and nitriding on martensitic DIN 16MnCr5 steel—are shown in Figure 2. The chemical composition mapping of nitriding layers with a thickness of ~8 μm and ~10 μm, showing the layer element in N-rich phases and the microstructure of DIN 16MnCr5 on the diffusion zone, is shown in Figure 2a. Studies found in the literature [15,16] suggest that the compound layer of the N-rich phase formed by γ’-Fe_4_N and ε-Fe_3_N has higher hardness and excellent wear resistance. The formation of a boriding layer with chemical composition elemental mapping of boriding layers with a thickness of ~70 μm and ~80 μm on DIN 16MnCr5 is shown in Figure 2b. It has been reported [23,26] that the boriding layer’s FeB and Fe_2_B phases increase the hardness and improve the wear resistance. The surface’s cross-section analysis by SEM and the elemental composition EDS mapping of layers formed by gas-nitriding and pack boriding layers with a thickness of ~80 μm and ~100 μm are shown in Figure 2c. Layers with the presence of N-rich and B-rich content can be observed.

**Figure 3:**
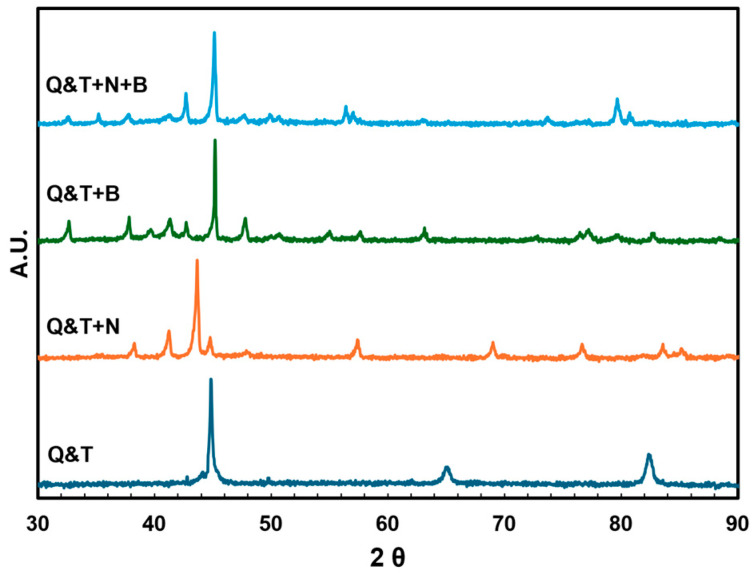
Diffraction XRD patterns for quenched and tempered DIN 16MnCr5 and surface treatments with nitriding and boriding.

The XRD analysis of DIN 16MnCr5 steel with thermal treatment of quenching and tempering at 870 °C for 15 min, diffusion treatments of gas-nitriding at 560 °C for 6 h, pack boriding at 950 °C for 4 h, and duplex gas-nitriding and pack boriding are shown in Figure 3. The main diffraction patterns for quenching and tempered DIN 16MnCr5 samples are α-Fe (PDF 01-087-0721) (110) at 44.67°, (200) at 65.02°, and (211) at 82.34° and negligibly transform to γ-Fe (PDF 00-052-0513) (111) at 42.75° and (200) at 49.78° due to the quenching and tempering treatment. After the gas-nitriding treatment of quenched and tempered DIN 16MnCr5 samples, the diffraction patterns were ε-Fe_3_N (PDF 04-007-3377) (110) at 38.32°, (002) at 41.3°, (111) at 43.75°, (112) at 57.60°, (300) at 69.3°, (113) at 77.01°, and (302) at 83.99°, as well as γ’-Fe_4_N (PDF 01-071-4924) (111) at 41.14°, (200) at 47.89°, (220) at 70.03°, and (311) at 84.57° as a function of temperature and nitriding potential described by the Lehrer diagram [29]. This indicates that the surface layer is mainly composed of ε-Fe_3_N and γ’-Fe_4_N, providing higher hardness and improved wear resistance [14,15,16,17,18,19,20]. The diffraction patterns for the pack boriding treatment of quenched and tempered DIN 16MnCr5 steel samples exhibit peaks corresponding to the main phases formed in the surface layer FeB (PDF 04-007-6176) (200) at 32.56°, (201) at 39.58°, (111) at 41.24°, (210) at 45.08°, (102) at 47.74°, (211) at 50.65°, (301) at 54.96°, (112) at 57.6°, (020) at 63.04°, (312) at 77.21°, (113) at 79.91°, (122) at 82.77°, Fe_2_B (PDF 04-019-2627) (200) at 36.38°, (002) at 43.67°, (211) at 46.59°, (112) at 51.26°, (202) at 58.1°, (310) at 59.15°, (213) at 82.32°, MnB (PDF 00-038-1424) (020) at 32.16°, (101) at 37.13°, (102) at 38.95°, (111) at 40.64°, (021) at 44.54°, (210) at 46.72°, (121) at 49.94°, (130) at 54.16°, (211) at 56.5°, (002) at 62.32°, and Cr_2_B (PDF 04-004-1242) (002) at 41.82°, (211) at 44.30°, (310) at 56.04°, and (213) at 78.11°, showing increased mechanical properties of microhardness and elastic modulus and thereby improving the wear resistance [23,24,25,26,27]. Finally, the diffraction patterns for duplex gas-nitriding and pack boriding were ε-Fe_3_N, γ’-Fe_4_N, FeB, Fe_2_B, MnB, and Cr_2_B. For nitriding and boriding patterns, the microstrain affects the width of the reflections but not their position in the diffractogram, as reported by Boca et al. [30].

**Figure 4:**
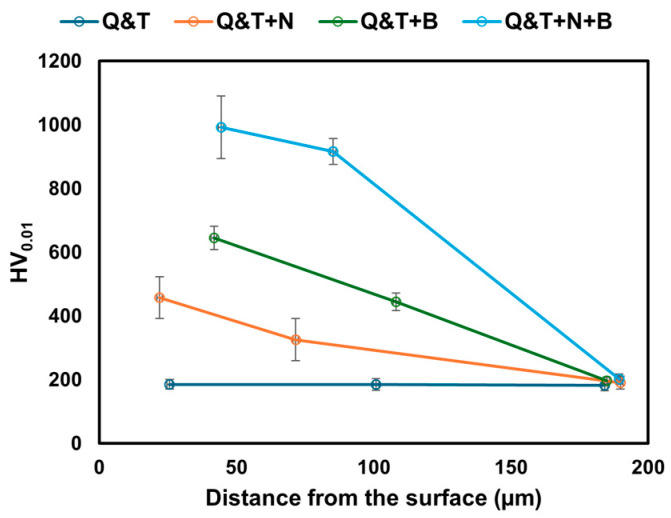
Microhardness profile as a function of a distance from the surface for quenched and tempered DIN 16MnCr5 and surface treatments with nitriding and boriding.

The average value of the microhardness measurement profile as a function of the distance from the surface for quenched and tempered DIN 16MnCr5 and surface treatments with gas-nitriding, pack boriding, and duplex gas-nitriding and pack boriding is shown in Figure 4. The microhardness profiles were measured from the cross-section of the surface towards the center of the samples, including the top surface or compound layer and the diffusion zone. For the sample surface of quenched and tempered DIN 16MnCr5, with an average of 184.1 HV, there are no microhardness variations between the top surface and the center for initial conditions. The microhardness for gas-nitriding treatment revealed an increase of 457.2 HV on the top surface, decreasing to the diffusion zone with 189.2 HV. Boriding treatment presented an increase of 644.9 HV on the top surface, decreasing to the diffusion zone with 197.4 HV. The duplex gas-nitriding and pack boriding treatment showed an increase of 992.3 HV on the top surface, decreasing to the diffusion zone with 201.8 HV. It has been reported by Caliari et al. [19] that the mechanisms for increasing the hardness of the diffusion nitriding zone are related to the interstitial solid solution of N in the matrix and the precipitation of nitrides. On the other hand, the increase in surface hardness with boriding is related to the formation of secondary phases MnB and Cr2B manganese in addition to the hard FeB and Fe2B iron borides and the formation of a solid solution of boron in iron, as reported by Calik et al. [23].

**Figure 5:**
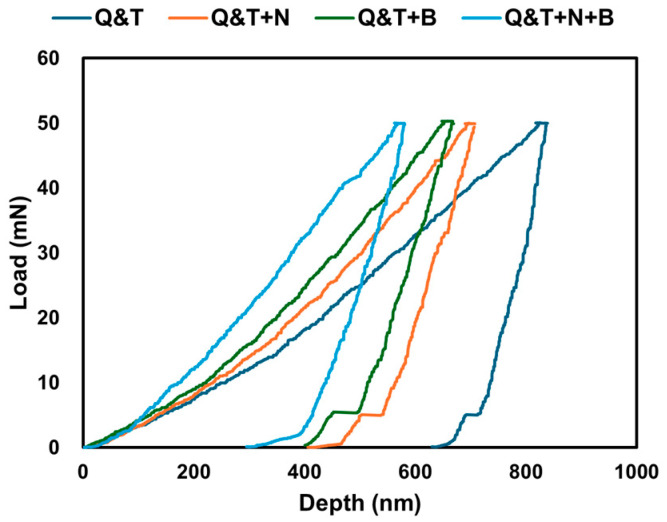
Nanoindentation load–unload curves for quenched and tempered DIN 16MnCr5 and surface treatments with nitriding and boriding.

The nanoindentation load–unload curves for quenched and tempered DIN 16MnCr5 and surface treatments with gas-nitriding, pack boriding, and duplex gas-nitriding and pack boriding are shown in Figure 5. The load–unload curves corresponding to quenched and tempered DIN 16MnCr5 have a maximum depth of 837.8 nm. In contrast, nanoindentation by the Berkovich method surface treatments with gas-nitriding, pack boriding, and duplex gas-nitriding and pack boriding showed a maximum depth of 705.6 nm, 667.4 nm, and 580.3 nm, respectively. It can be observed that there is a reduction in the maximum depth of duplex gas-nitriding and pack boriding. The mechanical properties of elastic modulus and nanohardness are shown in Table 4. The nanohardness and elastic modulus agree with the boriding condition reported by Calik et al. [27], reporting a nanohardness of ~2.7 GPa and 3.7 GPa and an elastic modulus of ~135 GPa and 159 GPa.

**Figure 6:**
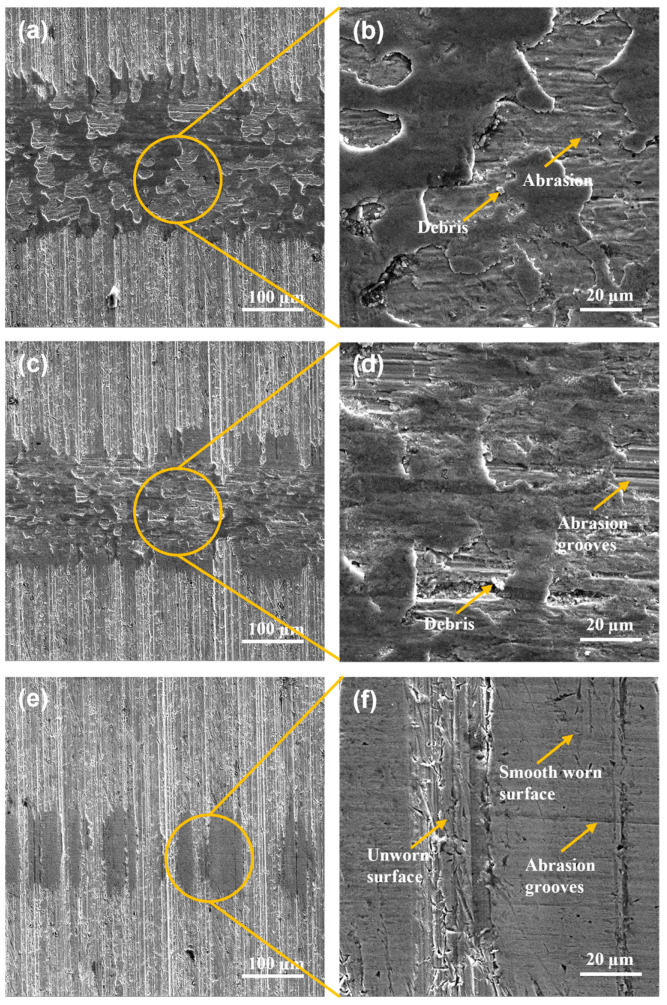
SEM micrographs with secondary electron detector wear tracks for Q&T DIN 16MnCr5 steel (**a**,**b**) with 6 N load, (**c**,**d**) with 4 N load, and (**e**,**f**) with 2 N load.

The SEM images of worn surfaces of DIN 16MnCr5 steel with thermal treatment of quenched and tempered treatment surfaces after the pin-on-disc tests performed using loads of 2 N, 4 N, and 6 N at 100 m of sliding distance under dry sliding conditions are shown in Figure 6. The width of the wear track can be seen in Figure 6a,c,d. It can be observed that the samples of 6 N and 4 N exhibit severe worn scratches with the formation of abrasion grooves as the main wear mechanisms, as can be seen in Figure 6b,d. The surface using 2 N reveals a smooth, worn surface with the presence of slight abrasion grooves and unworn zones due to a small load. As shown in micrographs, failure mechanisms in DIN 16MnCr5 steel surfaces, including debris agglomeration and abrasion, have been reported as the predominant wear modes [3,7,9].

**Figure 7:**
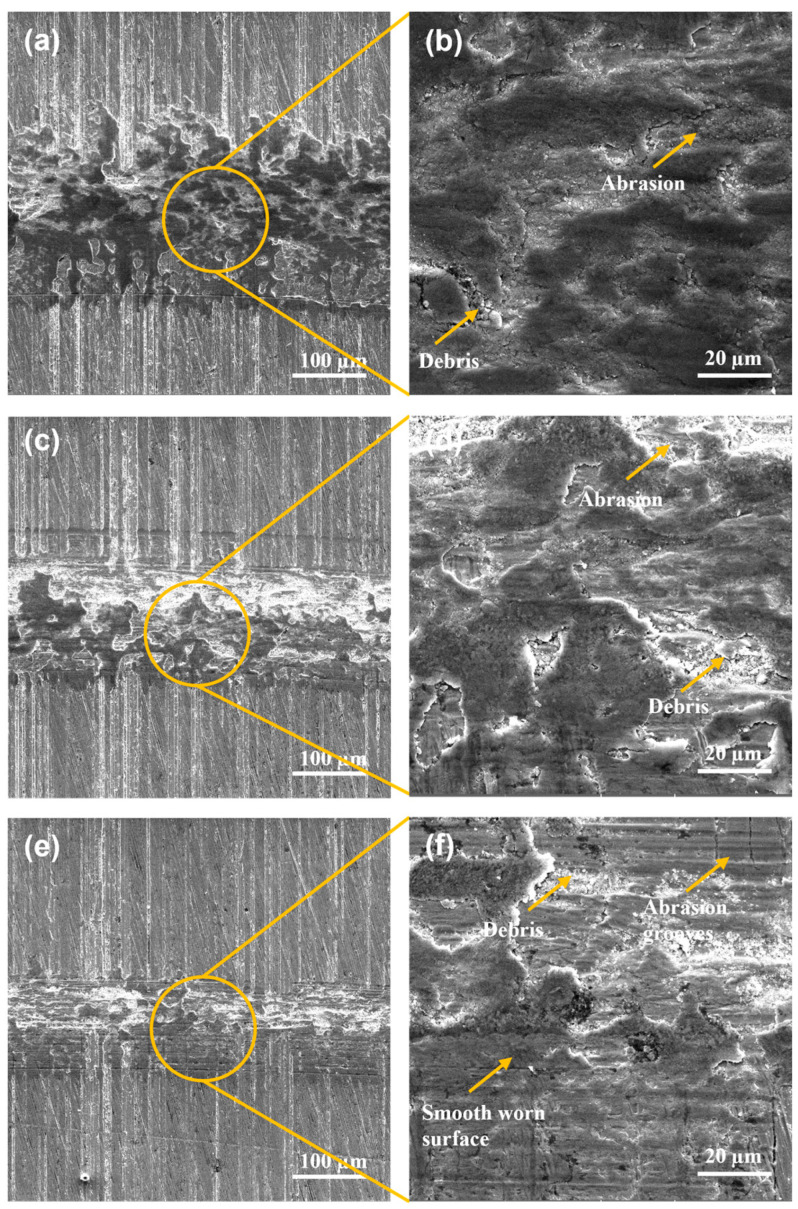
SEM micrographs with secondary electron detector wear tracks for Q&T+N DIN 16MnCr5 steel (**a**,**b**) with 6 N load, (**c**,**d**) with 4 N load, and (**e**,**f**) with 2 N load.

The damaged surfaces after the pin-on-disc tests performed on the wear track for the surfaces using quenching and tempering and gas-nitriding treatment of Q&T+N DIN 16MnCr5 steel samples are shown in Figure 7. The wear track widths presented a decrease for Q&T+N DIN 16MnCr5 steel samples due to the reduction in loads used, as shown in Figure 7a,c,d. The worn surfaces using 6 N and 4 N exhibited deformation as the dominant wear mechanism, as shown in Figure 7b,d. The worn surface, subjected to a load of 2 N, exhibits smooth, worn abrasion grooves and unworn zones due to the small load. The primary failure mechanisms for the nitriding surfaces were micro-cracks, debris agglomeration, and abrasion. Wong-Ángel et al. [16] have also observed similar failure mechanisms, such as material agglomeration, micro-cracks, and abrasion, as the predominant wear modes.

**Figure 8:**
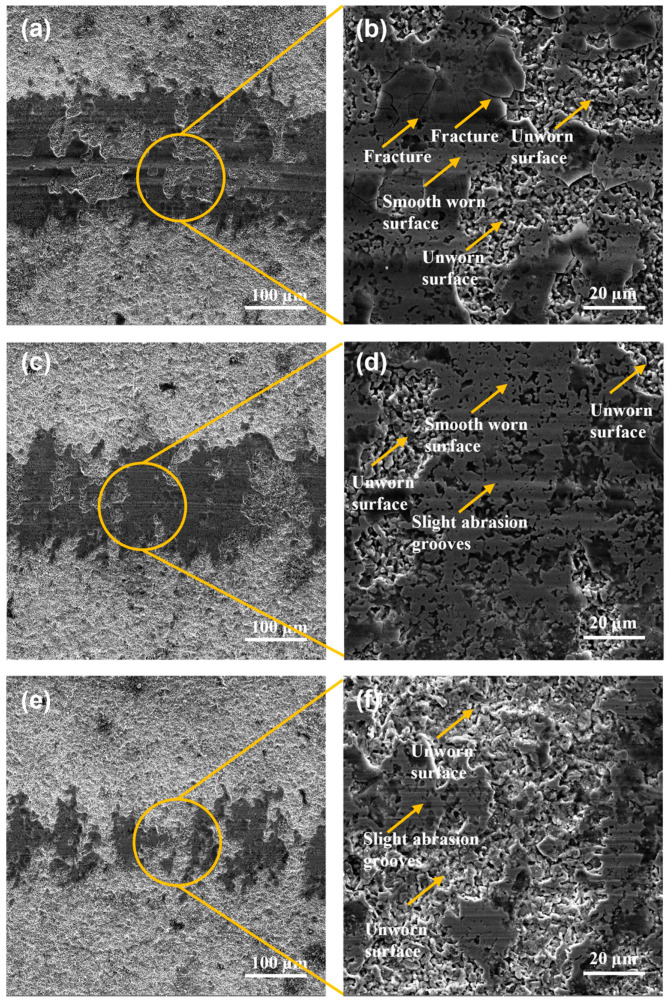
SEM micrographs with secondary electron detector wear tracks for Q&T+B DIN 16MnCr5 steel (**a**,**b**) with 6 N laod, (**c**,**d**) with 4 N load, and (**e**,**f**) with 2 N load.

The worn surfaces for gas-nitriding treatment of quenched and tempered Q&T+B DIN 16MnCr5 samples are shown in Figure 8. The width of the wear tracks was measured after the pin-on-disc tests were performed, showing the different magnitudes of damage according to the applied load shown in Figure 8a,c,d. The worn surfaces using 6 N and 4 N have deformation as the dominant wear mechanism, as shown in Figure 8b,d. The wear track surface with a load of 2 N reveals a smooth, worn surface with the presence of slight abrasion grooves, smearing, and unworn zones due to a small load, as shown in Figure 8f. The failure mechanisms for all the pack boriding samples were abrasion, debris agglomeration, and micro-cracks. Ulutan et al. [31] reported that boronized steel has a much higher abrasion wear resistance than untreated steel. In the present study, similar behavior can be observed with a reduction in the abrasion wear mechanism compared with untreated quenched and tempered DIN 16MnCr5 steel.

**Figure 9:**
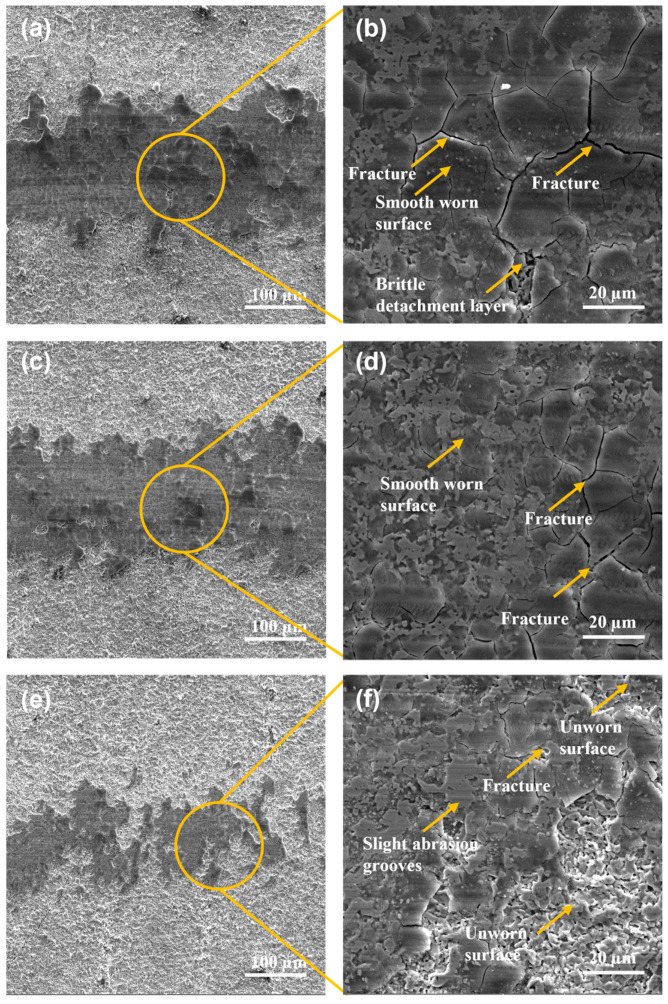
SEM micrographs with secondary electron detector wear tracks for Q&T+N+B DIN 16MnCr5 steel (**a**,**b**) with 6 N load, (**c**,**d**) with 4 N load, and (**e**,**f**) with 2 N load.

The wear tracks for duplex gas-nitriding and pack boriding treatments of Q&T+N+B DIN 16MnCr5 samples are shown in Figure 9. The widths of the wear track for the different loads were measured for Q&T+B DIN 16MnCr5 steel samples, as shown in Figure 9a,c,d. The worn surfaces using 6 N and 4 N showed evidence of fracture and spalling as the dominant wear mechanism, as shown in Figure 9b,d. The surface with a load of 2 N revealed a smooth worn surface with the presence of micro-cracks and slight abrasion grooves, smearing, and unworn zones due to the small load (see Figure 9f). The failure mechanisms for combined gas-nitriding and pack boriding treatments were abrasion, debris agglomeration, micro-cracks, and brittle detachment layers for all the pack boriding samples. Numerous studies have shown that treating surfaces with enhanced nitriding or boriding processes can improve their mechanical and tribological properties, thus altering their wear modes. Wong-Ángel et al. [16] observed micro-cracks and abrasion as the primary wear modes, while Ulutan et al. [31] identified abrasion as the predominant wear mode. Additionally, in this study, the surface exhibited minor abrasion grooves and layer fractures.

**Figure 10:**
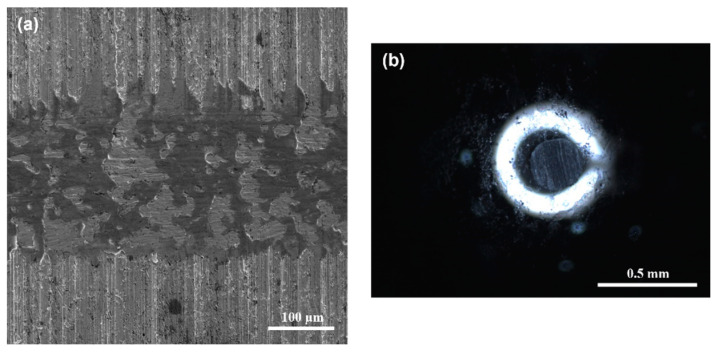
Worn surfaces of (**a**) Q&T DIN 16MnCr5 steel and the (**b**) WC pin with a 6 N load.

SEM images of worn surfaces of DIN 16MnCr5 steel with thermal treatment and quenching and tempering treatment and the WC pin (after pin-on-disc tests performed using a load of 6 N at 100 m sliding distance under dry sliding conditions) are shown in Figure 10. Figure 10a shows the wear track of the base material with evidence of an entirely worn surface. The WC pin shows the contact pair after pin-on-disc tests with evidence of wear, as shown in Figure 10b.

**Figure 11:**
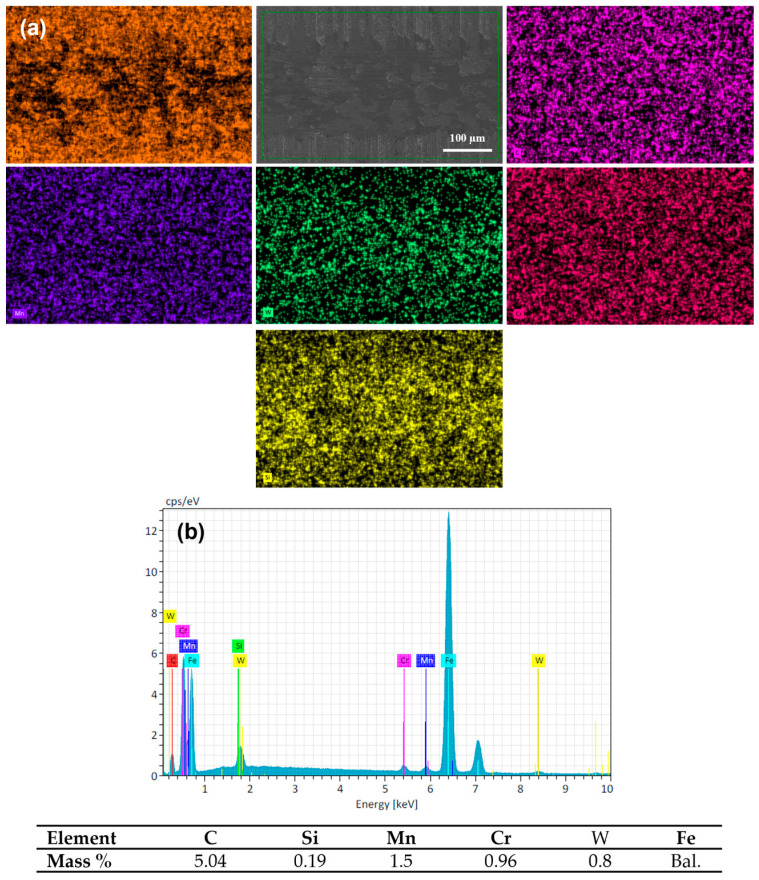
Chemical composition mapping of worn surface Q&T DIN 16MnCr5 steel (**a**) and energy-dispersive spectroscopy EDS (**b**) with 6 N load.

The SEM micrograph and the elemental composition EDS mapping analysis of the wear track surface of quenched and tempered 16MnCr5 steel are presented in Figure 11. The EDS mapping shows the presence of chemical elements of the base material DIN 16MnCr5 steel (see Figure 11a). Within this analysis, the presence of the transferred element of the pin by adhesion from the pin was determined by adding the element W. The semi-quantitative EDS analysis detected a small amount of W, suggesting slight adhesion as a wear mechanism due to the transfer of pin material on the wear surface (see Figure 11b). The adhesive wear mechanism could be caused by the limited mechanical properties of the quenched and tempered DIN 16MnCr5, which promotes soft mechanical counter-body contact.

**Figure 12:**
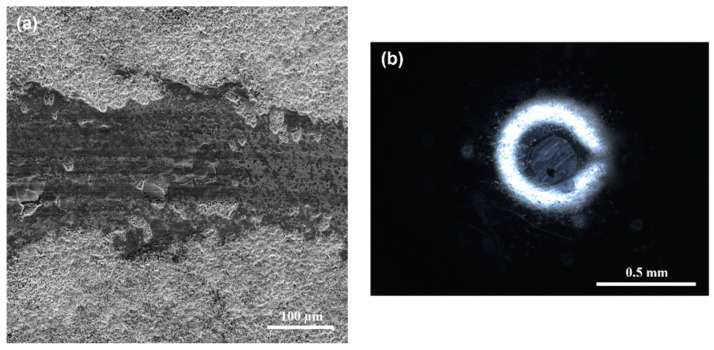
Worn surface of (**a**) Q&T+N+B DIN 16MnCr5 steel and (**b**) WC pin with 6 N load.

The SEM images of the worn surfaces of Q&T+N+B DIN 16MnCr5 steel and the WC pin are shown in Figure 12. These images were obtained after conducting pin-on-disc tests with a 6 N load and 100 m of sliding distance under dry sliding conditions. In Figure 12a, the wear track of the Q&T+N+B DIN 16MnCr5 steel displays a smooth surface with slight evidence of damage. On the other hand, Figure 12b shows the WC pin exhibiting wear after the pin-on-disc tests.

**Figure 13:**
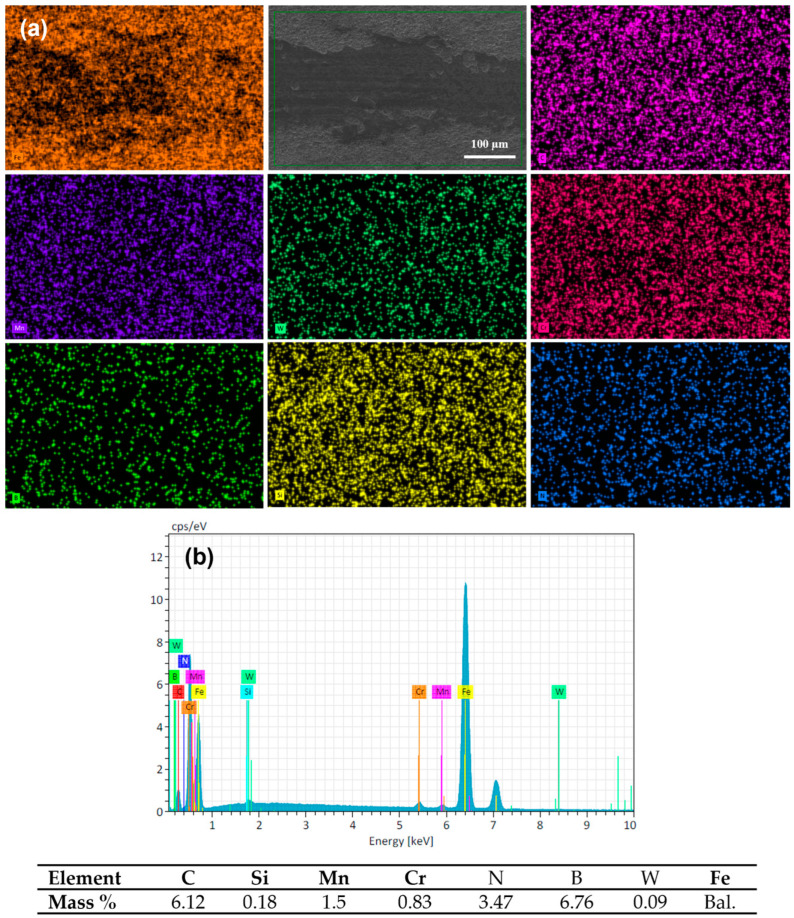
Chemical composition mapping of worn surface Q&T+N+B DIN 16MnCr5 steel (**a**) and energy-dispersive spectroscopy (EDS) (**b**) with 6 N load.

The wear track surface analysis by SEM and the elemental composition EDS mapping of layers formed by duplex gas-nitriding and pack boriding are shown in Figure 13. The presence of the base material DIN 16MnCr5 and layers with the presence of N-rich and B-rich content can be observed in the layer (see Figure 13a). Additionally, the W element was added to the analysis to determine the presence of a transferred element of the pin by adhesion from the pin. The amount of W was almost undetected in this analysis, which suggests that the wear mechanism was not altered by the transfer of pin material on the wear surface (see Figure 13b).

**Figure 14:**
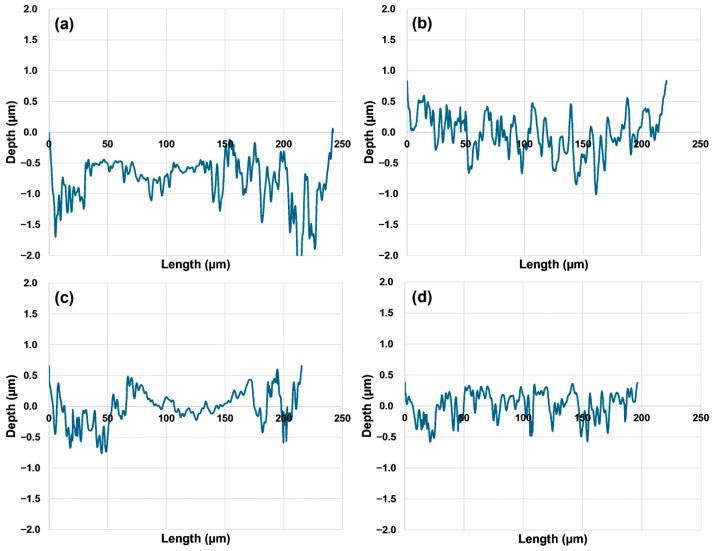
Cross-sectional profilometry of the worn surface for (**a**) Q&T, (**b**) Q&T+N, (**c**) Q&+B, and (**d**) Q&T+N+B.

Figure 14 shows the results of the worn surface cross-section of the pin-on-disc sliding test, performed under dry conditions with a load of 6 N and 100 m sliding distance, of quenched and tempered DIN 16MnCr5 steel alongside surface treatments with gas-nitriding, pack boriding, and duplex gas-nitriding and pack boriding. The loss volume was calculated by determining the area of the cross-sectional wear track using a numerical method and then multiplying it by the circular perimeter of the wear track. The worst damage to the cross-sectional depth of the wear track can be observed for the quenched and tempered DIN 16MnCr5 steel sample (see Figure 14a). The volume loss for the quenched and tempered DIN 16MnCr5 steel was 0.0107 mm^3^. The samples for gas-nitriding and the pack boriding conditions presented a slight reduction in damage, as shown in Figure 14b,c, compared to the quenched and tempered DIN 16MnCr5 steel. The calculated volume losses for gas-nitriding and the pack boriding samples were 0.0083 mm^3^ and 0.0074 mm^3^, respectively. Finally, the best condition that exhibited a reduced depth and length damage profile for wear tracks was duplex gas-nitriding and pack boriding, as shown in Figure 14d. The calculated volume loss for duplex gas-nitriding and pack boriding was 0.0042 mm^3^.

**Figure 15:**
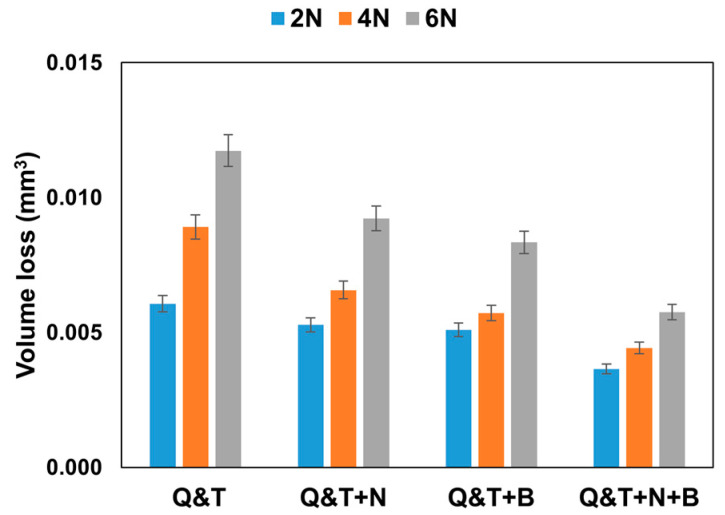
Volume loss results for Q&T, Q&T+N, Q&T+B, and Q&T+N+B.

The results of the volume loss from the pin-on-disc sliding test conducted under dry conditions using loads of 2 N, 4 N, and 6 N with a sliding distance of 100 m on quenched and tempered DIN 16MnCr5 and surface treatments such as gas-nitriding, pack boriding, and duplex gas-nitriding and pack boriding are presented in Figure 15. The results indicate that the quenched and tempered DIN 16MnCr5 steel exhibited the highest volumetric wear. Conversely, gas-nitriding and pack boriding showed greater wear resistance compared to the quenched and tempered DIN 16MnCr5 steel, resulting in reduced volumetric wear. This behavior aligns with findings in the literature, attributing the wear resistance to hardened surfaces influenced by compound layers and the diffusion nitriding zone [16,18,19] as well as the presence of iron borides and the formation of a solid solution of boron in iron [23,24,25,26]. In comparison, the surface treated with duplex gas-nitriding and pack boriding displayed the highest wear resistance due to the combined effect of enhanced mechanical properties, including increased hardness and elastic modulus.

**Figure 16:**
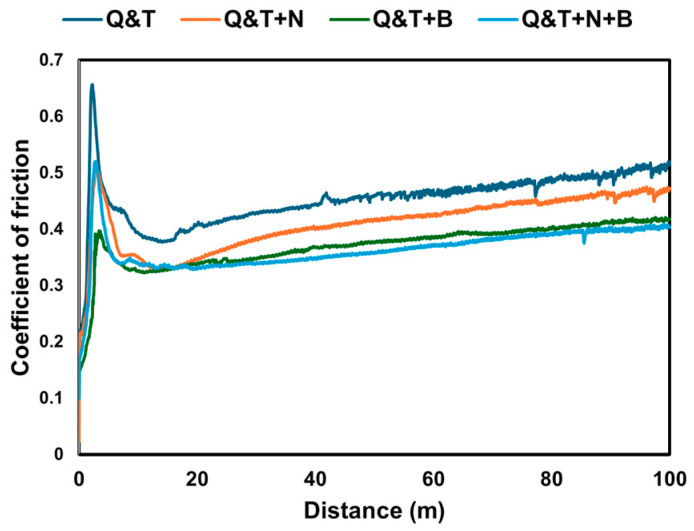
Coefficient of friction for quenched and tempered DIN 16MnCr5 and surface treatments with nitriding and boriding.

The graph in Figure 16 shows the coefficient of friction for tribological tests using 6 N of samples in relation to the sliding distance of quenched and tempered DIN 16MnCr5 steel and different surface treatments, including gas-nitriding, pack boriding, and duplex gas-nitriding and pack boriding. The average coefficient of friction values after running-in, from 20 m to 100 m, showed a decreasing trend, as follows: µ = 0.455 for quenched and tempered DIN 16MnCr5 steel, µ = 0.413 for gas-nitriding, µ = 0.376 for pack boriding, and the lowest value of friction, µ = 0.365, for duplex gas-nitriding and pack boriding when compared to quenched and tempered DIN 16MnCr5 steel. In general, the friction increased for the running-in samples up to 20m until stable behavior was reached. Afterward, there was a slight increase in the coefficient of friction trend throughout the test for quenched and tempered DIN 16MnCr5 and surface treatments with nitriding and boriding. The quenched and tempered DIN 16MnCr5 steel exhibited the highest friction values during the sliding wear test. In contrast, the coefficient of friction decreased by 9.18% for gas-nitriding, 17.27% for pack boriding, and 19.77% for duplex gas-nitriding and pack boriding compared to quenched and tempered DIN 16MnCr5 steel. These results show that surface treatments with nitriding and boriding have a significant influence on the coefficient of friction.

## 4. Conclusions

The microstructure, chemical composition, mechanical properties, and tribological behavior of quenched and tempered DIN 16MnCr5 steel as well as surface treatments with gas-nitriding, pack boriding, and duplex gas-nitriding and pack boriding were investigated. Based on the experimental results, the following main conclusions can be drawn.

- The nitriding layers of ~8 μm and ~10 μm were formed by γ’-Fe_4_N, and ε-Fe_3_N, showing the layer in N-rich phases and the microstructure of DIN 16MnCr5 steel in the diffusion zone. A boriding layer of ~70 μm and ~80 μm was formed by FeB and Fe_2_B iron borides on DIN 16MnCr5 steel. The duplex gas-nitriding and pack boriding layers of ~80 μm and ~100 μm showed the presence of N-rich and B-rich content in layers with γ’-Fe_4_N, ε-Fe_3_N, FeB, and Fe_2_B phases.
- The samples treated with gas-nitriding, pack boriding and duplex gas-nitriding, and pack boriding showed increased microhardness, elastic modulus, and nanohardness, resulting in higher wear resistance.
- The quenched and tempered DIN 16MnCr5 steel showed higher volumetric wear compared to the gas-nitriding and pack boriding processes, which exhibited 49% lower volumetric wear.
- Compared to quenched and tempered DIN 16MnCr5 steel, the coefficient of friction decreased by 9.18% for gas-nitriding, 17.27% for pack boriding, and 19.77% for duplex gas-nitriding and pack boriding.

## Figures and Tables

**Table 1:** Parameters for thermal, gas-nitriding, and pack boriding treatments.

Sample	Quenched and Tempered	Gas-Nitriding	Pack Boriding
Q&T	15 min/870 °C	-	-
Q&T+N	15 min/870 °C	6 h/560 °C (30% NH_3_ + 70% H_2_)	-
Q&T+B	15 min/870 °C	-	4 h/950 °C powder wt.% (90% SiC, 5% B_4_C, 5% KBF_4_)
Q&T+N+B	15 min/870 °C	6 h/560 °C (30% NH_3_ + 70% H_2_)	4 h/950 °C powder wt.% (90% SiC, 5% B_4_C, 5% KBF_4_)

**Table 2:** Chemical composition wt.% of commercial DIN 16MnCr5 steel.

Element	C	Si	Mn	P	S	Cr	Fe
	0.14–0.19	0–0.4	1.0–1.3	0–0.025	0–0.035	0.8–1.1	Bal.

**Table 3:** Tribological experimental test conditions.

Parameters	Dry Condition
Normal force (N)	2, 4, 6
Sliding speed (m/s)	0.1675, 0.1256, 0.0837
Sliding distance (m)	100
Environment	Air
Track diameter (mm)	16, 12, 8
Temperature (°C)	21
Humidity (RH)	40–60

**Table 4:** Mechanical properties from nanoindentation tests.

Sample	Nanohardness H (GPa)–HV	Elastic Modulus E (GPa)
Q&T	1.8–187.1	101.9
Q&T+N	3.2–332.4	141.7
Q&T+B	3.8–390.5	161.2
Q&T+N+B	8.7–887.1	194.5

## Data Availability

The original contributions presented in the study are included in the article, further inquiries can be directed to the corresponding author.
